# Supervised Speaker Diarization Using Random Forests: A Tool for Psychotherapy Process Research

**DOI:** 10.3389/fpsyg.2020.01726

**Published:** 2020-07-28

**Authors:** Lukas Fürer, Nathalie Schenk, Volker Roth, Martin Steppan, Klaus Schmeck, Ronan Zimmermann

**Affiliations:** ^1^Clinic for Children and Adolescents, University Psychiatric Clinic, Basel, Switzerland; ^2^Department of Mathematics and Computer Science, University of Basel, Basel, Switzerland; ^3^Division of Clinical Psychology and Psychotherapy, Faculty of Psychology, University of Basel, Basel, Switzerland

**Keywords:** supervised speaker diarization, psychotherapy process measure, dyadic audio analysis, EMRAI speech corpus, random forest

## Abstract

Speaker diarization is the practice of determining who speaks when in audio recordings. Psychotherapy research often relies on labor intensive manual diarization. Unsupervised methods are available but yield higher error rates. We present a method for supervised speaker diarization based on random forests. It can be considered a compromise between commonly used labor-intensive manual coding and fully automated procedures. The method is validated using the EMRAI synthetic speech corpus and is made publicly available. It yields low diarization error rates (M: 5.61%, STD: 2.19). Supervised speaker diarization is a promising method for psychotherapy research and similar fields.

## Introduction

Human interaction is organized by interpersonal coordination that manifests itself in temporally coordinated behavior. Interpersonal coordination can be broadly grouped into behavior matching and interpersonal synchrony, which involve the rhythmic and “smooth meshing of interaction” over time (Bernieri and Rosenthal, 1991). During the dyadic interactions of psychotherapy, patients and therapists have been shown to synchronize in verbal, non-verbal, and physiological behavior (Marci et al., 2007; Ramseyer and Tschacher, 2008; Lord et al., 2015; Koole and Tschacher, 2016; Kleinbub, 2017). A growing body of empirical research has associated the degree to which interpersonal synchrony is present during therapy with therapeutic outcome (Ramseyer and Tschacher, 2014), empathy (Marci et al., 2007; Imel et al., 2014; Lord et al., 2015), the formation of the therapeutic relationship (Ramseyer and Tschacher, 2011), personality traits (Tschacher et al., 2018), and emotion regulation (Galbusera et al., 2019; Soma et al., 2019). Due to their integrative value, processes of interpersonal synchrony have thus moved to the center of attention of psychotherapy research and related fields (Ramseyer and Tschacher, 2006). In the case of non-verbal movement synchrony, motion energy analysis has become a widespread tool to quantify movement from video (Ramseyer, 2013). It is made available through standalone software (Ramseyer, 2019), a MATLAB implementation (Altmann, 2013), and an R-package for synchronization analysis and visualization (Kleinbub and Ramseyer, 2019). This allows researchers to engage non-verbal synchrony in an automized, objective, reproducible, and non-labor-intensive fashion in their respective setting and has accelerated research on non-verbal movement synchrony in the clinical dyad (Delaherche et al., 2012). In the same line, autonomic measures (heart rate, skin conductance, breathing) applied in the field of interpersonal physiology (Kleinbub, 2017) also benefit from accessible measurement in the naturalistic setting (Weippert et al., 2010; Pijeira-Díaz et al., 2016; Barrios et al., 2019). In contrast, studies on vocal quality or vocal coordination have not gained the same amount of attention (Imel et al., 2014; Reich et al., 2014; Tomicic et al., 2017; Soma et al., 2019; Zimmermann et al., 2020). This is somewhat surprising because audio recordings are a widely used tool for educational, scientific, and supervisory activities (Aveline, 1992) and, in comparison to video or physiological measures, are non-invasive and inexpensive to attain in high quality. However, while the processing of non-verbal movement or physiological measures is facilitated through software solutions and devices, post-processing of audio for quantitative statistics can be strenuous due to speaker diarization (Anguera et al., 2012).

## Speaker Diarization in Psychotherapy Research

Speaker diarization is the practice of determining who speaks when (Anguera et al., 2012). In other words, diarization means creating a feature stream indicating speaker identity over time. Diarization in psychotherapy research is currently practiced in two different ways. On one side researchers rely on manual annotation of speaker identity, being time intensive but accurate (Imel et al., 2014; Reich et al., 2014; Soma et al., 2019). On the other side researchers rely on unsupervised automated methods, presenting with a minor work intensity but also with higher error rates (Xiao et al., 2015; Nasir et al., 2017a,b). The term “unsupervised” indicates that the system is not given prior knowledge as to how the speakers are embodied in the audio features. Mostly, the audio stream is segmented into speaker homogenous segments, which then are clustered (Tranter and Reynolds, 2006). In the field of psychotherapy research, studies have used unsupervised methods producing diarization error rates above 10%. For example, Xiao et al. (2015) used automatic speech recognition in motivational interviewing to produce text-based empathy scores of sessions and compare them with human empathy ratings. They employed a clustering based unsupervised diarization procedure that produced an error of 18.1%. Nasir et al. (2017b) predicted the outcome of couple therapy using speech features. The audio stream was segmented to indicate speaker changes based on generalized likelihood ratio criteria, which then are clustered to provide speaker-homogenous segments. Average pitch information in these segments are then used to provide a speaker annotation (wife or husband). They report a diarization error rate of 27.6%. While fully automated diarization procedures are appealing, diarization error rates can substantially be improved when introducing a learning step into the procedure, based on a small quantity of pure data (Sinclair and King, 2013). This relates to the idea of supervised machine learning. A recent study on a new fully supervised speaker diarization method using recurrent neural networks reported an error rate of 7.6% on a corpus of telephone calls (Zhang et al., 2019).

As described, regarding diarization practices in psychotherapy research, researchers tend to rely either on manual coding, which makes research very cost intensive, or they resort to fully automized unsupervised methods. In order to overcome this obstacle and to accelerate scientific undertakings on audio recordings in psychotherapeutic settings, we introduce a method for supervised speaker diarization, developed to work for standard single microphone audio recordings of dyadic talk psychotherapies. Considering the workload, the supervised method is a compromise between work intensive manual annotation and error prone unsupervised methods. It involves creating a learning set and introducing a learning step prior to automatically diarizing the whole data set.

## Aim of This Study

The aim of this study is to present a supervised method for dyadic speaker diarization based on a random forest algorithm. The method is tested using a freely available speech corpus. In the future, this will allow testing alternative methods and refinements of the current method on the same data set. The code has been made publicly available (Fürer, 2020). The procedure has been aggregated to one function and the preparations to run the function have been documented. We hope that this allows researchers with minimal coding experience or unfamiliar with MATLAB to carry out analyses on their own. The method is conceptualized in MATLAB and relies on readily available components (Segbroeck et al., 2013; Giannakopoulos and Pikrakis, 2014). We hypothesize that the method will produce diarization error rates comparable to current supervised diarization methods employed in other fields (below 10% per dyad; Zhang et al., 2019). Based on using random forest algorithm, we further hypothesize that the dyadic out-of-bag error rate (explained below) will positively correlate with the dyadic diarization error calculated on a test set. In future studies, this would allow quality checks on a dyadic level without producing a separate test set.

## Methods

### Random Forest

The presented method for supervised speaker diarization in dyadic psychotherapy is based on a random forest algorithm. While machine learning methods in general have gained attention in psychological research (Orrù et al., 2020), random forests can be considered a rather understandable machine learning algorithm that has already found its way into psychotherapy research (Imel et al., 2015; Masías et al., 2015; Husain et al., 2016; Sun et al., 2017; Wallert et al., 2018; Zilcha-Mano, 2019; Rubel et al., 2020; Zimmermann et al., 2020). The random forest algorithm is a machine learning classifier based on decision trees (Kotsiantis, 2013). The random forest combines a certain amount of decision trees in a single prediction model and is consequently also called an ensemble learner. It can be employed for regression or classification problems. When confronted with classification problems, the decision is a majority vote over all trees in the ensemble, which, in ensemble format, provides greater accuracy (Breiman, 2001). Major advantages of the random forest algorithm are that it is insensitive to multicollinearity in the input data and to variables that do not contribute to the classification strength (Imel et al., 2015). In our setting this is of importance since we don’t know which variables will be important for which dyad, and it is assumed that speech features may be highly correlated. The “random” in random forest refers to the usage of a random subsample of variables and a random subsample of data entries in the learning set when growing each tree (Husain et al., 2016). The process of randomly selecting a subsample of data entries without replacement for the training of each tree is called bagging (Breiman, 2001). This bagging process allows for the calculation of an out-of-bag error rate, which can be considered an estimate for the generalization error (Breiman, 1996). For each entry in the learning set the trees not using this specific entry for learning can be identified. They are called the out-of-bag classifier. The out-of-bag error is the error produced by the out-of-bag classifier, estimated using only the learning set. Given our use case, the possibility to estimate the generalization error with only the learning set is useful: If we apply this method to new and real psychotherapy audio and calculate the out-of-bag error on the learning data, we can estimate the overall strength of the prediction in each dyad, informing us for which dyads the diarization worked well and for which it didn’t. We therefore report the correlation of the dyadic out-of-bag error with the dyadic speaker error (explained below) calculated in the separate test set.

### Supervised Diarization in Dyadic Psychotherapy

The dyadic nature of talk therapy allows for an assumption to simplify the otherwise more complicated diarization process: the number of speakers is known, two in this case. Relating to the idea of supervised learning, here, a classifier is given prior knowledge as to how the two speakers are embodied in the input features (supervised diarization). Fortunately, inside the context of psychotherapy research, the classifiers do not have to be generalizable to different dyads, but rather, multiple classifiers can be trained, each one specialized to diarize one dyad only. The necessary steps involve: (1) creation of a learning set for each dyad (human coder), (2) automatic silence detection, (3) automatic voice activity detection, (4) feature extraction, (5) learning to provide a dyadic classifier, (6) prediction in one dyad, and (7) data aggregation. The steps are explained below.

### EMRAI Synthetic Diarization Corpus

The supervised diarization method is tested on the EMRAI Synthetic Diarization Corpus (Edwards et al., 2018). This corpus is based on the LibriSpeech Corpus (Panayotov et al., 2015), namely recordings of English audiobooks. The manual labeling of audio data for training purposes is extremely time intensive. Thus, the authors of the corpus have synthetically created both 2-person and 3-person “dialogues” with and without overlap by sequentially arranging spoken parts. The EMRAI synthetic diarization corpus thereby offers an opportunity for testing diarization systems built for the context of the dyadic conversations as given in talk therapy.

### Silence Detection and Voice Activity Detection

For silence detection, an algorithm calculates an individual intensity threshold value for each session recording. For more information, please refer to the source code (Fürer, 2020). The result of silence detection is a vector indicating silence and non-silence windows in the audio file. In a second step, voice activity detection is performed using a robust and competitive voice activity detection system for MATLAB developed by Segbroeck et al. (2013). This differentiates between voice and noise in the non-silence windows. Voice activity detection was performed over the whole audio, not only in non-silence windows. The procedure feeds contextually expanded spectral cues related to speech (spectral shape, spectro-temporal modulations, harmonicity, and the spectral variability) to a standard Multilayer Perceptron classifier (Segbroeck et al., 2013).

### Feature Extraction

In order to allow the classifier to accurately differentiate between patient and therapist speech, appropriate features need to be extracted from the audio file. We aimed at using an existing and open source MATLAB library to make the procedure replicable by others. Features are provided by the MATLAB Audio Analysis Library and its function “stFeatureExtraction” (Theodoros and Aggelos, 2014). The function yields a total of 35 audio features: energy, zero-crossing rate, entropy of energy, two spectral centroids, spectral entropy, spectral flux, spectral flux roll-off, 13 Mel-frequency coefficients, 12 chroma vectors, harmonic ratio, and mean fundamental frequency. All audio features and their calculations are described in detail in the introductory publication accompanying the library (Giannakopoulos and Pikrakis, 2014). Here, we will focus our description on the Mel-frequency coefficients (MFCCs), since they are crucial features for speaker diarization (Friedland et al., 2009). The calculation of MFCCs takes into account that our perception of the frequency spectrum is not linear (Goldstein, 2010). We perceive differences in lower frequencies as more predominant than differences in higher frequencies. This non-linear relationship is represented by the mel scale, a function which, informed by psychoacoustics, mimics the human auditory system (Zhou et al., 2011). First, the audio signal is represented in the frequency domain by calculating the log discrete Fourier transform. The power spectrum then is submitted to a mel-scale filter bank consisting of overlapping triangular bandpass filters. Their bandwidth and spacing are given by a linear mel scale interval (Umesh et al., 1999). That way, the frequency spectrum is filtered (warped) in the same way, as it is thought to be filtered in the auditory system. MFCCs are then provided as the discrete cosine transform of the mel-filtered log power spectrum, providing coefficients in the time scale (Kathania et al., 2019). The authors of the MATLAB Audio Analysis Library have calculated MFCCs according to Slaney (1998).

In addition to the features provided by the MATLAB Audio Analysis Library, we calculated HF500, being a voice quality ratio between high spectral energy (above 500 Hz up to 3500 Hz) and low spectral energy (80–500 Hz). It has extensively been used in arousal quantification from speech (Bone et al., 2014; Chen et al., 2016).

All features, including silence and voice activity detection, are calculated in non-overlapping windows of 0.1 s in width, and all features have been used in training and predicting the diarization models.

### Learning and Classification

The described features are then used to train a random forest classifier per dyad to predict speaker identity based of the features using the available speaker annotations from the learning set. Only spoken parts (labeled with person-1 or person-2 speech, no silence) were introduced to the learning set. To illustrate, the learning set would contain the timestamps (start of utterance and stop of utterance) and a variable of speaker identity of all utterances for person-1 and person-2 in the first 10 min of the recording. Using the corresponding features, an ensemble of 500 trees is trained for each dyad, using Breiman’s algorithm (Breiman, 2001). All EMRAI dialogues of length bigger than 20 min (*n* = 107) were selected. The first 10 min of each dialogue were chosen for learning purposes, while minutes 10–20 were used as a test set. This simulates the creation of a learning set in a naturalistic setting. Using 10 min of audio in the learning material means that each speaker is represented by less than 5 min of speech (Mean: 4.17 min, Std: 0.38). After training, the classifier is then used to predict speaker identity in the independent test set (minutes 10–20 of the respective recording), resulting in classifications of either person-1-speech or person-2-speech. Please note that the classifier would also yield a decision for actual silence windows; it was not trained to discriminate between silence, noise, and spoken parts. This requires aggregating information to a final decision.

### Data Aggregation

After classification is acquired, three information streams must be aggregated in order to produce a final diarization vector. Results of silence detection (silence or no silence), voice activity detection (voiced or unvoiced), and random forest based diarization (person-1 or person-2 speech) are combined to a feature stream of 0.1s segments of either non-speech, person-1-speech, or person-2-speech according to the following rules: Windows classified as silence by the silence detection remain unchanged. Non-silence windows, however, are replaced by the information stream of the voice activity detection resulting in a combined stream indicating silence, non-speech/noise, and speech. The windows classified as speech are then replaced by the person-1-speech and person-2-speech labels obtained by the respective classifier. The resulting vector contains the labels “non-speech” (silence or noise), “person-1-speech,” and “person-2-speech.”

### Error Reporting and Data Set

The performance of a speaker diarization method is assessed via the diarization error rate (Barras et al., 2006), a measure comprised of the sum of the following elements: (1) *speaker error* (SpE, percentage of times the wrong speaker is predicted), (2) *missed speech* (MSp, percentage of times silence is predicted instead of speech), (3) *false alarm speech* (FASp, percentage of times speech is predicted instead of silence), and (4) *overlap error* (percentage of times overlapped speech is not assigned to one of the respective speakers). Given our choice of using 2-person non-overlapping speech, the diarization error rate (DER) is reported as the sum of the first three errors (Reynolds and Torres-Carrasquillo, 2005). SpE, MSp, and FASp are reported as mean values with standard deviations over all dyads, same-sex dyads, and different-sex dyads. The sampling frequencies (fs) of the corpus and our prediction stream were different (fs corpus = 100, fs prediction stream = 10) insofar as 10 windows at a time of the corpus are summarized to match one window of our prediction. Transitional windows, where more than one classification was present in the corpus windows to be summarized (both speech and silence), are excluded from the analyses.

We also hypothesized that the dyadic out-of-bag error would be a useful measure to control for the quality of the diarization (speaker annotation) in any specific dyad. We report the correlation between the dyadic out-of-bag error and the dyadic SpE.

## Results

### Total DER, Speaker Error, Missed Speech, False-Alarm Speech

Table 1 provides an overview over error rates. Although total mean DER can be considered low, there are differences between dyads, as already implied by higher error rates for same sex dyads than different sex dyads, *t*(61) = 4.16, *p* = 1.01e-04. While the error produced through silence detection and voice activity detection (MSp + FASp) seems to show high stability throughout dyads (Mean: 3.11, Std: 1.27), SpE is more prone to vary over dyads (Mean: 2.50, Std: 2.12). This is confirmed by the correlation of the total DER and the SpE, *r*(105) = 0.83, *p* = 1.24e-28. This implies that the variability in total DER is mainly produced by the SpE. Forty of 107 dyads presented a total DER below 5%. Ninety-one of 107 dyads had a total DER below 7.5%. Five dyads showed total DER above 10%. Hence there are dyads for which the method had somewhat increased DER (16 dyads with DER above 7.5%). Mean FASp error rates are below 1%, mean MSp error rates are located just above 2%.

**TABLE 1:** Mean *error rates* (Std) in percent over all dyads (*n* = 107), same-sex dyads (*n* = 44), and different-sex dyads (*n* = 63).

	**Total DER**	**Speaker Error**	**MSp**	**FASp**
All dyads	5.61 (2.19)	2.50 (2.12)	2.60 (1.07)	0.51 (0.88)
Same-sex dyads	6.48 (2.57)	3.60 (2.58)	2.47 (0.98)	0.41 (0.75)
Different-sex dyads	5.01 (1.64)	1.74 (1.28)	2.69 (1.13)	0.57 (1.04)

**FIGURE 1:**
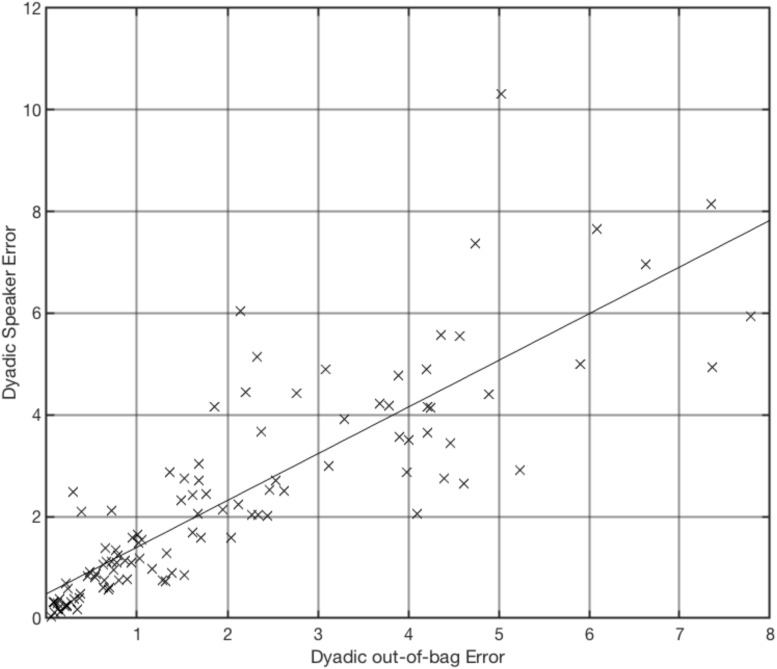
Dyadic speaker error (e.g., speaker-1 predicted instead of speaker-2 and vice versa) in percent against dyadic out-of-bag error over all dyads (*n* = 107), *r*(105) = 0.85, *p* = 1.65e-30.

As expected, the dyadic out-of-bag error did correlate positively with the dyadic SpE, providing evidence for the usefulness of the out-of-bag error to estimate the quality of the diarization for specific dyads (*r*(105) = 0.85, *p* = 1.65e-30, see Figure 1).

## Discussion

Speaker diarization in psychotherapy research has both been performed in a manual, time-consuming (Imel et al., 2014; Reich et al., 2014; Soma et al., 2019), and, alternatively, unsupervised, automated fashion (Xiao et al., 2015; Nasir et al., 2017a,b). For certain scientific contexts, a supervised procedure can be favorable, as it greatly reduces effort (manpower, time, and costs) compared to manual diarization and yields low error rates. In this study, we have described a method for supervised speaker diarization feasible for the dyadic nature of talk therapy. The method requires that the user manually creates a learning set of approximately 5 min cumulative length per speaker. A random forest classifier is trained from the learning set, one for each dyad, using speech features extracted by the MATLAB Audio Analysis Library (Giannakopoulos and Pikrakis, 2014). The classifier is then set out to diarize the whole amount of data (sessions) of this respective dyad. The distinction between voiced and unvoiced windows is made using an already existing procedure for voice activity detection by Segbroeck et al. (2013) and a custom silence detection algorithm. The method is made publicly available (Fürer, 2020). A major advantage of the study is that an open source speech corpus was used to present first results of the proposed method. The availability of the corpus allows other researchers to present results of other methods on the same data set or allows the test of the impact of improvements to the here proposed method.

The method shows satisfying diarization error rates (Mean: 5.61, Std: 2.19), comparable to other fully supervised methods (Zhang et al., 2019). Error rates are higher for same sex dyads (Mean: 6.48, Std: 2.57) than for different sex dyads (Mean: 5.01, Std: 1.64), *t*(61) = 4.16, *p* = 1.01e-04. This result is expected. The classifier is faced with features of higher degree in similarity when dealing with same-sex dyads, resulting in higher error rates. The difference of diarization error in those groups is mainly due to speaker error (confusion of the classifier toward the distinction of speaker one and speaker two). Speaker error and the total diarization error correlate with *r*(105) = 0.83, *p* = 1.24e-28, indicating that the total diarization error is mainly produced by speaker error, while missed speech and false alarm speech errors are more stable across dyads. While false alarm speech rates are substantially low (below 1%), missed speech rates are located around 2.5%. Low false alarm speech rates reflect the additional use of silence detection, which has shown to be very robust in differentiating silence windows from non-silence windows. *Post hoc* analyses for silence detection over the whole test set (all dyads together) reveal a miss rate (non-silence predicted instead of silence) of 0.70% and a false alarm rate (silence predicted instead of non-silence) of 1.51%. Considering the synthetic nature of the corpus used in this study, where the audio files of the corpus contain only silence or speech (no noise), voice activity detection may seem needless besides silence detection. In an environment, where one can be sure that no noise occurs (only speech or silence), the sole use of silence detection can be considered favorable. For later use of the method on naturalistic data, however, where noise may well be part of the equation, voice activity detection is indispensable and is therefore introduced as well. Both silence detection and voice activity detection have been incorporated in the code published (Fürer, 2020).

For 16 (out of 107) dyads, total diarization error exceeds 7.5%. When working with real psychotherapy data, it would be practical to be able to identify these dyads without creating a separate test set. Therefore, we tested whether the out-of-bag error presents a good estimate for the dyadic speaker error. The correlation showed to be high, *r*(105) = 0.85, *p* = 1.65e-30. We argue that the out-of-bag error can be used to make assumptions toward the quality of diarization, maybe leading to the exclusion of specific dyads, for reasons of error management. It is encouraged for future research to include the out-of-bag error as moderator variable to control for noise.

## Limitations and Challenges

In comparison to manual annotations of speaker identity, unsupervised and supervised procedures of speaker diarization will be error prone. It is therefore important for future studies of this realm to report how and to what extent diarization errors influence the research findings at hand. As we reported, the out-of-bag error can be used for this purpose. However, there are no clear guidelines, for example, indicating the need to exclude a dyad for reasons of untolerable diarization error. Consequently, researchers are encouraged to at least publish diarization error rates and to test whether study results correlate with the diarization errors found.

Further, applying machine learning methods to psychotherapeutic data involves experience in programming. Proximity to data scientific or machine learning colleagues is not always guaranteed for workgroups invested in psychotherapy research. It was therefore important to us to publish the code used in this study (Fürer, 2020). The procedure is summarized to one function and an extensive explanation of preprocessing steps is given, in order to make it applicable by users with minimal coding experience.

While using a speech corpus may allow testing future improvements, future studies should invest in testing the proposed procedure on real psychotherapy data in order to clarify concerns toward the validity of results. For an application example of the procedure we refer the reader to the study of Zimmermann et al. (2020), which is using the method presented here to analyze the impact of silence across speaker switching patterns in psychotherapy sessions. Dyadic out-of-bag errors were comparable to the errors found here (Mean: 5.3%, Std = 3.3).

In light of the growing interest in interpersonal processes in psychotherapy, the supervised diarization applied in the study at hand may facilitate the exploration of dyadic vocal and conversational processes that may be linked to change processes, treatment outcome, diagnoses, and patient characteristics. Also, it may facilitate process research to uncover trajectories of variables of interest based on audio recordings. By catalyzing studies concerned with speech and conversational measures, psychotherapy research will gain in rater-independent, objective measures that can widely be used by various research groups and thereby provide results that are comparable and reproducible.

## Data Availability Statement

The datasets generated for this study are available on request to the corresponding author.

## Author Contributions

LF, RZ, and VR contributed to the conception, method, and design of the study. LF performed the analysis and wrote the manuscript with support and revisions of NS, MS, KS, and RZ. All authors contributed to manuscript revision, read, and approved the submitted version.

## Conflict of Interest

The authors declare that the research was conducted in the absence of any commercial or financial relationships that could be construed as a potential conflict of interest.
